# Veterans walk to beat back pain: study rationale, design and protocol of a randomized trial of a pedometer-based Internet mediated intervention for patients with chronic low back pain

**DOI:** 10.1186/1471-2474-11-205

**Published:** 2010-09-13

**Authors:** Sarah L Krein, Tabitha Metreger, Reema Kadri, Maria Hughes, Eve A Kerr, John D Piette, Hyungjin Myra Kim, Caroline R Richardson

**Affiliations:** 1 VA Ann Arbor Health Services Research and Development Center of Excellence, VA Ann Arbor Healthcare System, Ann Arbor, MI, USA; 2 Department of Internal Medicine, University of Michigan Medical School, Ann Arbor, MI, USA; 3 Center for Statistical Consultation and Research, University of Michigan, Ann Arbor, MI, USA; 4 Department of Family Medicine, University of Michigan Medical School, Ann Arbor, MI, USA

## Abstract

**Background:**

Chronic back pain is a significant problem worldwide and may be especially prevalent among patients receiving care in the U.S. Department of Veterans Affairs healthcare system. Back pain affects adults at all ages and is associated with disability, lost workplace productivity, functional limitations and social isolation. Exercise is one of the most effective strategies for managing chronic back pain. Yet, there are few clinical programs that use low cost approaches to help patients with chronic back pain initiate and maintain an exercise program.

**Methods/Design:**

We describe the design and rationale of a randomized controlled trial to assess the efficacy of a pedometer-based Internet mediated intervention for patients with chronic back pain. The intervention uses an enhanced pedometer, website and e-community to assist these patients with initiating and maintaining a regular walking program with the primary aim of reducing pain-related disability and functional interference. The study specific aims are: 1) To determine whether a pedometer-based Internet-mediated intervention reduces pain-related functional interference among patients with chronic back pain in the short term and over a 12-month timeframe. 2) To assess the effect of the intervention on walking (measured by step counts), quality of life, pain intensity, pain related fear and self-efficacy for exercise. 3) To identify factors associated with a sustained increase in walking over a 12-month timeframe among patients randomized to the intervention.

**Discussion:**

Exercise is an integral part of managing chronic back pain but to be effective requires that patients actively participate in the management process. This intervention is designed to increase activity levels, improve functional status and make exercise programs more accessible for a broad range of patients with chronic back pain.

**Trial Registration Number:**

NCT00694018

## Background

Chronic pain, especially low back pain, is a significant problem worldwide, with one fourth of adults in the U.S. reporting low back pain in the past three months and about one-half reporting back pain during a given year [1-3]. Low back pain is generally considered chronic when it persists for longer than three months [4], and the longer the pain persists the greater the risk for long-term disability [5]. Chronic back pain affects both younger and older adults with potentially significant consequences regardless of age. Among younger adults, chronic back pain is associated with disability, unemployment and lost productivity, whereas for older adults chronic back pain is associated with functional limitations, economic difficulty and social isolation [5-7]. Chronic pain-related conditions are among the major drivers of healthcare costs in the U.S. and chronic low back pain is the single most costly condition in terms of work loss [8].

Pain affects many patients using the U.S. Department of Veterans Affairs (VA) health care system [9-12], and may be even more prevalent in VA than in the general population. Fifty to seventy percent of VA general medicine patients suffer from chronic pain, defined as pain persisting for six or more months [9,11], with back pain the most commonly reported type of pain [13]. Back pain is also one of the most prominent complaints among Veterans returning from the conflicts in Iraq and Afghanistan [14] and the number of VA users with a low back pain diagnosis appears to be rising [15].

### Management of Chronic Back Pain

The high prevalence, high cost and negative consequences of chronic back pain underscore the significant need for effective and efficient approaches for managing this condition. Strategies for managing chronic back pain range from traditional medical management approaches (e.g., pharmacotherapy) and self-management programs to interventional pain management techniques and complementary and alternative medicine [16-22]. There is little, if any, sound evidence to support the use of surgery or other invasive interventions for most patients with chronic low back pain [4,23,24] and relatively strong evidence to support the effectiveness of exercise therapy, intensive multidisciplinary pain programs, and certain psychological interventions [16,19,22,25].

### Exercise and Chronic Back Pain

Staying active and exercise therapy can prevent recurrence, reduce pain, improve functional status, and decrease disability for patients with chronic back pain [16,19,21,26-28]. A meta-analysis by Hayden and colleagues [17] suggested that the most effective strategy for improving back pain consisted of an individually designed exercise program that included home-based supervision and a relatively intensive exercise regimen. Yet, the design, delivery and evaluation of an exercise program that incorporates these "ideal" components have yet to be accomplished.

Research also shows that a variety of exercise programs including yoga as well as aerobic and strengthening exercises result in both clinically and statistically significant improvements in outcomes for patients with chronic back pain [16,21,26-28]. However, most of the interventions studied have been short term, with outcomes measured within the first six months. While such studies may be appropriate for testing the efficacy of exercise among patients with chronic back pain under ideal circumstances, the intensity and required resources for these programs are likely to limit their sustainability and potential reach in real-world practices [17,21,28,29]. Thus, we still lack efficient and effective strategies for promoting exercise therapy among patients with chronic back pain or ways to use exercise as part of a treatment program [13,30-32].

A recent review article found only low to moderate evidence supporting walking as an effective intervention for low back pain [33]. Yet, the article also highlights the paucity of research in this area, with only four studies identified for the review and three of the four judged as being of poor methodological quality. The authors therefore conclude that further research on the role of walking as a primary intervention for managing chronic low back pain is needed.

### Conceptual Framework

**Figure 1:**
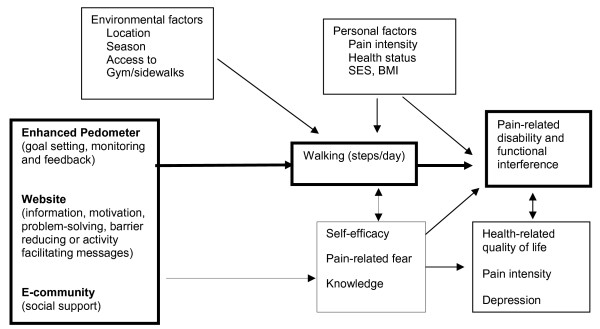
**Study conceptual framework**.

Given the challenges just described, we have developed an intervention that uses generally available tools and a relatively low cost approach to help patients with chronic back pain initiate and maintain an exercise program. The conceptual framework shown in Figure 1 summarizes the components of the intervention and how it is expected to influence pain-related disability or functional interference as well as other health outcomes. The intervention has three general components, each of which is described below along with the supporting rationale. First, the intervention uses an enhanced pedometer to promote walking directly through feedback, goal setting and monitoring. Walking is considered an ideal exercise since it is something that most anyone can do regardless of their health condition and does not involve the use of specialized equipment. Second, this intervention promotes walking through the use of a website that includestargeted content to enhance exercise self-efficacy. The website also contains materials to reinforce other important activities for managing chronic back pain, such as the use of strengthening and stretching exercises. The third component of the intervention is an e-community to provide social support, both peer and professional, that encourages patients to initiate and adhere to a walking program. We believe that more walking will contribute to improvements in function among patients with chronic back pain through multiple mechanisms, including improvements in mood, weight loss and by combating pain-related fear and self-protective behaviors, which can lead to reduced muscle tone, increased pain perceptions and functional disability.

### Key Intervention Components

#### Pedometers and walking programs

Others have previously demonstrated the feasibility and safety of home-based walking programs for individuals with chronic health conditions, including chronic pain conditions [34-38]. Additionally, studies suggest that a majority of middle-aged and older adults prefer physical activity outside a formal setting [39], and that participation rates and maintenance of physical activity are generally better in supervised home-based programs [17,40]. The use of pedometers has become increasingly popular both as a way for individuals to monitor step counts and as an objective tool used by researchers to assess physical activity [41]. Pedometer-based walking programs can increase walking, at least in the short term, across a range of patient populations, including those at high risk for cardiovascular disease [36] and patients with mental health conditions [42,43]. Few studies, however, have examined the sustainability or effectiveness of a pedometer-based intervention on a longer-term basis or assessed the use of "enhanced" pedometers [43]. Whereas a simple pedometer provides instant feedback in the form of daily step counts, an enhanced pedometer provides detailed tracking of walking duration, automated step count logging and can be linked to a computer-based system to assist with goal setting and feedback on goal attainment. Goal setting and self-monitoring are key components in the process of self-regulation [44,45]. Facilitating achievable performance accomplishments through goal setting and feedback, reinforcing successful self-care activities and providing positive feedback when someone makes an effort to change their behavior are also part of skill development and enhancing self-efficacy, which are both key constructs of Social Cognitive Theory [44,46].

#### Internet-mediated interventions

Internet based programs are an increasingly popular option for delivering behavior change interventions [18,47-49]. The Internet provides a flexible, low cost communication vehicle that can play a key role in physical activity interventions by facilitating the exchange of information and by delivering encouraging or motivational messages to a large number of participants. A web-based platform can be used to deliver either tailored or static messages that target important social-cognitive processes such as fear avoidance or self-efficacy. Self-efficacy, which is the level of confidence in one's own ability to perform a task or specific behavior [46], has been linked to the initiation and maintenance of physical activity [50-52] and a number of desired outcomes including better health-related quality of life or better physical functioning even when physical symptoms may not significantly improve [53-55].

#### E-communities and social support

One of the challenges of Internet-based behavior change programs is keeping participants interested and engaged for a sufficient duration, which can be important to ensure a therapeutic benefit [56]. An e-community is one potentially promising strategy for enhancing participation rates and improving the effectiveness of the intervention [18,56]. An e-community is a way for participants to both send and receive messages and can be developed using e-mail, threaded forums (asynchronous messaging) or chat rooms (synchronous, real-time messaging). The primary purpose of the e-community is to provide peer and/or professional support, which is strongly associated with physical activity and other health behaviors [51,52]. Receiving social support can have a positive effect on health and behavioral outcomes [57], self-efficacy [53], and physical activity [58]. Providing support to others also can lead to improved health behaviors on the part of the helper [59] as well as improved health outcomes and function [60]. Although the most commonly studied method of enhancing social support is through group programs, many patients face difficulties attending regular face-to-face meetings or group-based activities [45]. In a healthcare system in which resources are limited and patients face geographic and other barriers to accessing services, traditional group programs may also place a significant burden on patients and the health system. In contrast, e-communities, can translate the underlying principles and benefits of social support into systems that are effective for large, diverse patient populations.

## Methods/Design

**Figure 2:**
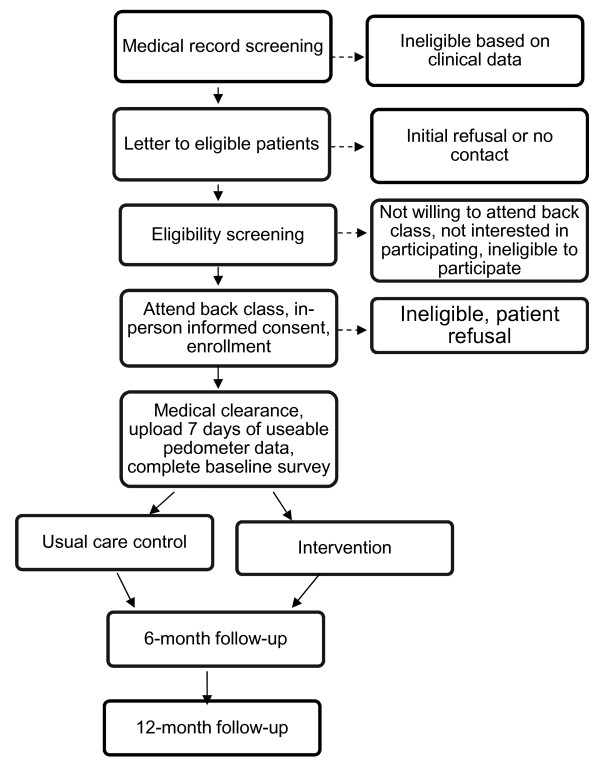
**Flow chart describing study recruitment and enrollment process**.

This randomized controlled trial includes outcomes measured at baseline, 6 and 12 months (Figure 2). The goal is to investigate the efficacy of the intervention in assisting patients with chronic back pain with initiating and maintaining a regular walking program. The primary outcome is pain-related disability and functional interference. The study specific aims are:

**Aim 1 **To determine whether a pedometer-based Internet-mediated intervention reduces pain-related functional interference among patients with chronic back pain in the short term and over a 12-month timeframe.

**Aim 2 **To assess the effect of the intervention on walking (measured by step counts), quality of life, pain intensity, pain related fear, and self-efficacy for exercise.

**Aim 3 **To identify factors associated with a sustained increase in walking over a 12-month timeframe among patients randomized to the intervention.

Patients with chronic back pain receiving care at a VA health care system are being recruited to participate in the study and are randomized to either enhanced usual care (control) or the pedometer-based Internet-mediated intervention. All participants are required to attend a general educational program ("back class") for individuals with chronic back pain. This ensures that all participants have a basic understanding of back mechanics and general strategies for managing back pain. Ethical approval for this study has been granted by the VA Ann Arbor Healthcare System human studies sub-committee.

### Eligibility Criteria and Identifying Eligible Patients

Potential participants are identified both through provider referral to back class and through data obtained from the VA electronic medical record system. Specifically, these data are used to identify patients who had two or more outpatient encounters in the prior 12 months with a diagnosis of back pain with no neurologic findings (ICD-9-CM codes 724.2, 724.5, 846.0-846.9). To be eligible for the study participants must: 1) have back pain that has persisted for more than 3 months; 2) have a sedentary lifestyle (less than 150 minutes of physical activity per week); 3) have access to a computer on at least a weekly basis with an available USB port and Internet access; 4) be able to provide written informed consent; 5) be able to communicate in English; 6) be non-institutionalized; 7) be able to walk at least one block; and 8) report they are not pregnant. Prior to participation, eligible patients must attend back class and obtain medical clearance from a physician, which may be their primary care provider, cardiologist, physical medicine and rehabilitation or pain management physician.

### Back Class

During back class, a physical therapist provides general education about back problems and strategies for managing back pain, with an emphasis on the importance of exercise (strengthening, stretching and aerobic) [materials available upon request]. During the class, patients practice strengthening and stretching exercises under the guidance of a physical therapist who adapts the exercises based on a patient's medical conditions. Up to 20 patients are scheduled per class, and each class takes approximately two hours.

### Patient Enrollment and Randomization

During the enrollment process, written informed consent is obtained and patients complete a six-minute walk test. All participants then receive an enhanced pedometer and USB cable to allow them to upload their step counts to a secure study website. Participants receive general guidance about how to use the pedometer and specific instructions for uploading data. The pedometer used for this study is the Omron HJ-720ITC, which contains a dual axial accelerometer, an internal clock, enough memory to store 42 days of detailed time stamped step count data, and an embedded USB port. The Omron pedometer is accurate within 1 to 4% compared to directly observed step-counts [61]. Pedometers that use accelerometers to detect motion are more accurate than spring and magnet pedometers when used by obese participants [62] and can be worn in different places on the body (as a necklace, on the hip, in a shirt pocket, in a pants pocket). For each hour of the day, the pedometer records a time and date, total steps, bout steps (10 minutes or more of continuous movement), and an activity flag that indicates if the pedometer detected any movement at all during the hour. During enrollment, study participants are also provided with instructions (including a log-in name and password) for accessing the study website to complete surveys or report adverse events.

Following enrollment, participants are instructed to wear their pedometer for seven days with the read out on the pedometer covered. At the end of the seven days they are asked to upload their pedometer data for the first time so we can establish a baseline measurement. After completing a baseline survey, uploading seven days of useable pedometer data and receiving medical clearance the participant is flagged as active in the system, and the computer randomly assigns them to the intervention or control group. The participant then receives an e-mail message with their group assignment, instructions to upload on either a weekly (intervention) or monthly (control) basis, and is instructed to remove the sticker covering the pedometer face.

### Enhanced Usual Care

Participants randomized to the control group, receive monthly e-mails asking them to upload their pedometer data. Those data are stored but not used for any further feedback to patients. Although the pedometer is an enhancement to what patients receive in standard practice, past studies suggest that pedometers in isolation are unlikely to result in a sustained increase in walking among generally sedentary individuals [43]. While control group participants are provided with log-on information for the study website, their access is limited to completing study surveys and reporting adverse events.

### Pedometer-Based, Internet Mediated Intervention

**Figure 3:**
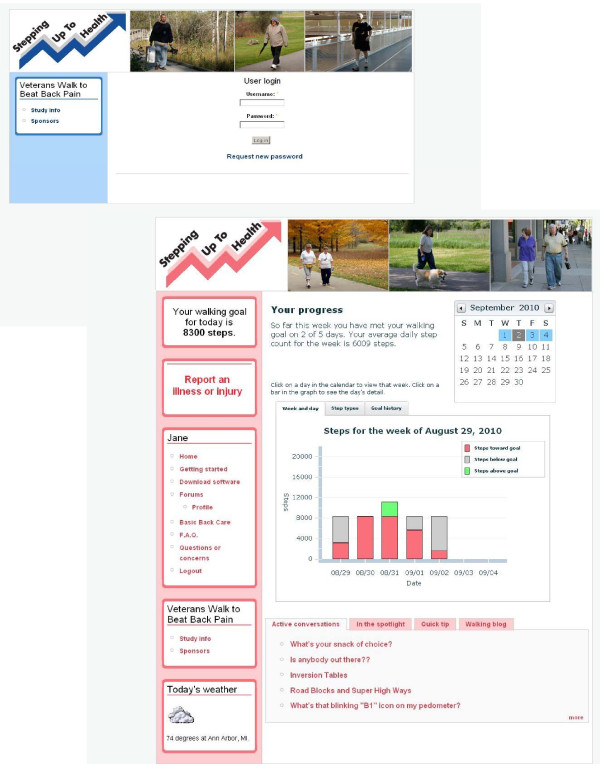
**Screen shots of study website**. The screen shots show the login page, the graphical and text feedback provided to an intervention participant and a list of active e-community topics. The screen shots also show the tailoring by gender, with variations in color and the photos that are displayed.

The study intervention is based on the Stepping Up to Health program developed by Richardson and colleagues [63]. The intervention consists of three primary components (Figure 1). Active intervention participants receive weekly e-mail reminders to upload their pedometers, weekly individualized walking goals and full access to the study website. Specifically, each person in the intervention receives computer generated personal walking goals. The goals are sent to participants by e-mail on a weekly basis and are automatically adjusted to assist the individual with increasing his/her step count at a reasonable pace or help with countering negative thoughts and behaviors when a particular goal is not achieved. Besides receiving a new goal each week, participants receive graphical and written feedback about their progress toward their goals when they log into the study website (Figure 3). The graph shows a breakdown of steps by day, week and month, while the text informs the participant about their average step count for the week and how many days his or her goal has been met up until that day. For example, it might read, "You have met your walking goal on 3 of 7 days. Your average daily step count for the week is 3568." While the general intent is for the total step goals to increase over time and thus promote an increase in walking, because the goals are established based on the participant's own activity level the goals can also be lower from one week to the next. Resetting a goal at a lower level can be beneficial when trying to counter the potential negative reaction and sense of failure for participants who may not be achieving the higher goals or for participants who might need to be reminded to start low and go slow after a relapse or health event that may have led to a decreased step count in the prior period. Participants who are not uploading data weekly receive step goals based on the last time they uploaded and will have the same goal until new activity takes place.

The participant website is tailored according to gender and includes information and messages (e.g., motivation, time constraints) to promote walking and a healthy lifestyle. New motivational or informational messages addressing potential barriers to physical activity (e.g., weather) or issues related to diet or other health conditions (e.g., cardiovascular disease, diabetes) appear every other day. Brief updates about topics in the news or new research findings related to back pain or physical activity are generally posted on a weekly basis so the website content does not get stagnant. The website also includes the materials and hand-outs that participants received at back class as well as a video demonstrating the proper technique for the strengthening and stretching exercises taught during the class.

The e-community component of the intervention consists of a thread-based forum that allows participants to conduct asynchronous text-based discussions. Each participant selects a pseudonym in order to establish an on-line identity while maintaining privacy. Following work by Lorig et al. [18], the e-community is used to provide both peer and professional social support. The e-community allows intervention participants to post messages, which can include asking questions, making suggestions or sharing success stories. The messages are monitored by research staff, including a physical therapist who does not provide individual medical advice but can respond to questions or back pain related issues with general information based on the VA low back pain guidelines. The forum also serves as a venue for generating competitions among the intervention participants to encourage walking and meeting walking goals. An advantage of an electronic forum is that it can prevent e-mail overload and all postings can be monitored to prevent inappropriate messages or communication. Experience to date shows that inappropriate messages are rare.

### Monitoring and Reporting of Adverse Events

The study includes several mechanisms for monitoring, troubleshooting and identifying the minority of patients for whom a relatively low intensity walking program is considered unsafe. Medical clearance is required and patients for whom walking is not a recommended activity, due to for example balance problems or cognitive impairment, are not eligible to participate. However, since we are trying to use relatively few exclusion criteria it is possible that some patients will be eligible but later develop signs or symptoms that suggest walking may no longer be a safe activity. Both intervention and control participants are encouraged to report adverse events including any health problems they experience whether or not they are thought to be study related. Participants can report adverse events by using a link on the website, by e-mail and over the phone. All email messages contain a link to the adverse events reporting form and the study's toll-free number for reporting adverse events. After completing four weeks of the study and every eight weeks thereafter, participants are prompted to complete a survey on the website that asks about specific adverse events and symptoms. This information is closely monitored by project staff so that any patients who appear to be experiencing a potentially serious health-related problem can be contacted for further assessment and follow-up.

### Description of Measures and Data Collection Procedures

Measures have been selected to assess our principal hypothesis that the proposed intervention will increase walking, as measured by step counts, among intervention patients relative to controls and reduce pain-related functional interference. Potential mediating factors, such as self-efficacy, and the benefits of a walking program, such as improvements in mood and quality of life, are also of significant interest. Data collection includes a computer-based survey completed at baseline, 6 months and 12 months, a six-minute walk test conducted at baseline and 12 months, and the step counts collected when participants upload their pedometer data. We are also collecting quantitative information about patients' use of the system via computerized records that the system maintains of all contacts. Health services use is assessed by patient report and by data obtained from the VA computerized medical record system.

The primary outcome for this study is pain-related disability and functional interference. Following the recommendations made through the Initiative on Methods, Measurement, and Pain Assessment in Clinical Trials (IMMPACT) [64], we are using both a disease-specific measure, the Roland and Morris Back Pain Disability Questionnaire (RDQ) index, and a generic measure of pain-related functioning from the medical outcomes study [65]. We hypothesize that the intervention will decrease back pain-related disability and pain-related functional interference. The RDQ index is a 24-item scale that has been widely used in back pain studies as a measure of self-perceived disability. The scale has good internal consistency, discriminative validity and is sensitive to change [66-69]. The medical outcomes study (MOS) pain measurement instrument assesses the effect of pain on mood and behaviors as well as the severity of pain over the past 4 weeks [65]. There is limited information about how the MOS instrument compares with other pain assessments but it is easily understood by patients and produces scales that have relatively good internal consistency [65].

Secondary outcomes include average daily steps, functional status, general health-related quality of life and pain intensity. Walking, which is a key aspect of the intervention, is measured as the number of average daily steps using the Omron HJ-720ITC pedometer. Rather than having to rely on self-reported step counts, the Omron allows us to upload the objectively recorded pedometer data directly to a database through the use of an embedded USB port. All data are time stamped by the pedometer. As an objective measure of function and to help validate the step count data obtained using the study pedometers we are conducting a six minute walk test at baseline and 12 months [70,71]. Participants are instructed to walk as far as they can in six minutes without running or jogging, with the primary measurement being the distance covered during those six minutes. Patients' general physical and mental functioning is being measured using the SF-12^® ^Health Survey [72] and pain intensity is evaluated using a numeric rating scale with standardized anchors (0 = "no pain" and 10 = "worst pain imaginable"), as used in the VHA's Pain as the Fifth Vital Sign initiative [73]. Patients use this scale to rate their current level of pain and their average level of pain during the past four weeks.

Other secondary outcomes and potential moderators include pain-related fear or kinesiophobia, which is being measured using a revised version of the Tampa Scale of Kinesiophobia (TSK-R) [74]; self-efficacy for exercise, which is being measured using the Exercise Regularly Scale [75]; the presence of depressive symptoms, which is assessed using a ten-item version of the Center for Epidemiologic Studies Depression Scale (CES-D 10) [76]; and, socio-demographic characteristics, such as age, gender and education level, other health conditions (e.g., diabetes), body mass index, perceived social support, health services utilization and employment status. Finally, an administrative interface to the website and database allows the study staff to track participant progress as well as ascertain whether a participant has been uploading step count data. This database will be used to assess participants' use of the different intervention components. For example, we will know how often the participant uploads step counts, how often they log on to the website, and whether they participate in the e-community.

### Analysis Plan

The patient is the primary unit of analysis for this study. Our sample size calculation was based on the RDQ index as the primary endpoint with a minimally detectable effect size determined as a difference of 0.4 standard deviations (SD) based on published data [68,77,78]. To detect a difference of 0.4 SD with 80% power using a two-sided 0.05 level two-group t-test, we will enroll 130 subjects in each group, to allow for an attrition rate of 30% at one year and a sample with outcome data of 100 patients in each group.

To determine whether the intervention reduces pain-related functional interference we will compare the difference in the RDQ index at 12 months between the two groups using a linear mixed-effects model. Similarly, mixed-effects models will be used to assess the effect of the intervention on step counts, quality of life, pain intensity, pain related fear and self-efficacy for exercise. Identifying factors associated with a sustained increase in walking over a 12-month timeframe is an exploratory analysis within the intervention group.

In addition, to understand features of successful versus less successful intervention experiences, we will analyze data collected through our monitoring of participant system use and semi-structured interviews conducted with a purposeful sample of intervention participants (choosing a sample of eight who expressed high satisfaction with the intervention and/or improved walking and a sample of eight with poor satisfaction and/or outcomes). Using standard qualitative methods [79], we will identify central themes from these interviews associated with the success of the intervention.

The two major limitations of the study design are: (1) we will be unable to directly compare the effect of this intervention to the effects of other types of exercise programs that have demonstrated promise in randomized trials (e.g., Yoga); and (2) usual care patients will receive some intervention elements (e.g., they will receive a pedometer and some of the educational content). Nevertheless, we believe that the design represents the optimal compromise between scientific rigor and real-world practicality. Further, we expect that neither the pedometer nor the extra education will have a substantial influence on our primary outcome. If they do have any impact, the effect would be to reduce the likelihood of finding treatment differences between the control and intervention groups. Thus, the design for this study will provide rigorous evidence about the effectiveness of the proposed intervention, which is novel and has yet to be evaluated and determined to be effective in isolation, as well as information about possible implementation in other settings.

## Discussion

Current evidence suggests that physical activity is one of the most effective strategies for managing chronic back pain and improving function [16,19,25]. Nonetheless, levels of physical activity are low among all segments of the U.S. population and especially individuals with chronic pain [80,81]. Despite the positive results of exercise observed in clinical trials little is known about how to effectively and efficiently assist patients with chronic conditions, and specifically those with unique challenges such as chronic back pain, with becoming more physically active and continuing to remain active over time. Given the positive benefits of exercise in managing chronic back pain and the significant need for effective strategies for increasing physical activity, the primary objective of this study is to assess the efficacy of a novel Internet and pedometer-based intervention that is designed to increase walking among individuals with chronic back pain, and thereby reduce pain-related disability and functional interference. Our hypothesis is that this intervention will increase activity levels, improve adherence to walking as needed to maintain the positive effects over time, and make exercise programs more accessible to a broad range of patients with chronic back pain.

## Competing interests

The authors declare that they have no competing interests.

## Authors' contributions

SLK, JDP, EAK, HMK and CRR were involved in the conception and design of the study. TM, RK and MH are involved in study data collection. All authors were involved in drafting the manuscript and revising it for critically important content. All authors have read and approved the final manuscript.

## Pre-publication history

The pre-publication history for this paper can be accessed here:

http://www.biomedcentral.com/1471-2474/11/205/prepub
